# Assessing the Dynamics and Control of Droplet- and Aerosol-Transmitted Influenza Using an Indoor Positioning System

**DOI:** 10.1038/s41598-019-38825-y

**Published:** 2019-02-18

**Authors:** Timo Smieszek, Gianrocco Lazzari, Marcel Salathé

**Affiliations:** 1 Modelling and Economics Unit, National Infection Service, Public Health England, London, UK; 2 MRC Centre for Outbreak Analysis and Modelling, Department of Infectious Disease Epidemiology, Imperial College School of Public Health, London, UK; 3 Center for Infectious Disease Dynamics, The Pennsylvania State University, University Park, USA; 4 Global Health Institute, School of Life Sciences, Ecole Polytechnique Fédérale de Lausanne (EPFL), Lausanne, Switzerland

## Abstract

There is increasing evidence that aerosol transmission is a major contributor to the spread of influenza. Despite this, virtually all studies assessing the dynamics and control of influenza assume that it is transmitted solely through direct contact and large droplets, requiring close physical proximity. Here, we use wireless sensors to measure simultaneously both the location and close proximity contacts in the population of a US high school. This dataset, highly resolved in space and time, allows us to model both droplet and aerosol transmission either in isolation or in combination. In particular, it allows us to computationally quantify the potential effectiveness of overlooked mitigation strategies such as improved ventilation that are available in the case of aerosol transmission. Our model suggests that recommendation-abiding ventilation could be as effective in mitigating outbreaks as vaccinating approximately half of the population. In simulations using empirical transmission levels observed in households, we find that bringing ventilation to recommended levels had the same mitigating effect as a vaccination coverage of 50% to 60%. Ventilation is an easy-to-implement strategy that has the potential to support vaccination efforts for effective control of influenza spread.

## Introduction

Despite extensive clinical experience and decades of research on influenza, it is still not fully understood how influenza is transmitted among humans. Traditionally, influenza transmission has been assumed to occur through the air, physical contact between humans, and by touching contaminated surfaces (i.e., fomites)^1,2^. Airborne transmission can occur in two ways: either through relatively large particles of respiratory fluid (droplets; 10^1^–10^2^ *μ*m) or through smaller such particles that can remain aerosolized (droplet nuclei; ≪10^1^ *μ*m)^3–7^. As larger droplets are pulled to the ground by gravity quickly, droplet transmission requires close physical proximity between infected and susceptible individuals, whereas aerosolized transmission can occur over larger distances and does not necessarily require that infected and susceptible individuals are at the same location at the same time^7^.

Until recently, close contact transmission was considered to be the dominant transmission pathway, largely because the evidence to support the importance of transmission through aerosols was mixed^1,7^. However, the question of the importance of the various transmission routes has received renewed attention recently, and multiple studies have in the past few years provided evidence for the importance of aerosol transmission^8–14^. There is increasing evidence from experiments with mammalian hosts that airborne transmission is much more efficient than fomite transmission^10,15^. Data from randomized controlled trials of hand hygiene and surgical face masks in households provided evidence that aerosol transmission accounts for half of all transmission events^13,14^. A nosocomial influenza outbreak with subsequent airflow analysis provided further evidence for the important role of aerosol transmission^11^. An experimental laboratory study using a patient examination room containing a coughing manikin provided further support for aerosol transmission^12^. A recent study with outpatients who tested positive for influenza A virus demonstrated that 53% and 42% produced aerosol particles containing viable influenza A virus during coughing and exhalation, respectively^16^. A mathematical model of influenza transmission within a household has suggested that the aerosol transmission route may not only be important, but indeed dominant^8^. Another mathematical model suggests that aerosol transmission is the dominant mode of transmission in long-term epidemics, whereas larger droplets could play a dominant role for short-term epidemics with high attack rates^5^.

Given the increasing evidence supporting an important role of aerosol transmission, it is prudent to revisit our expectations on disease dynamics of influenza outbreaks, and the best measures to control the spread of influenza. To address this issue, we use a high-resolution dataset of a medium-sized US high school, where both individual’s indoor positions and close proximity contacts to others were measured using wireless sensor network technology and perform computation simulations on it.

## Results

**Figure 1:**
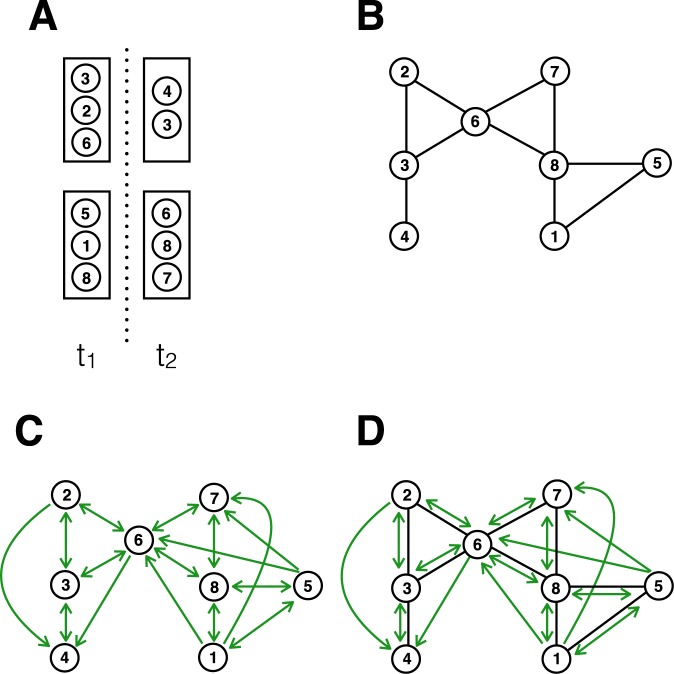
Simplified scheme of transmission routes. Different sets of individuals in the school can occupy the same rooms, at different times t1 and t2 (**A**). In these rooms, individuals may be in close proximity to one another. For visual simplicity, we assume in this figure that all individuals in the same room at the same time are in close proximity (but note that in the model, proximity is given by the sensor measurements). In the aerosol model, infected individuals can shed infectious material while in the room, which in turn may infect those individuals in the room concurrently or later on. The network of possible transmission pathways will therefore look different depending on the transmission routes. Based on the spatio-temporal pattern shown in panel (A), panel (B) shows the network of pure droplet transmission; panel (C) shows the network of pure aerosol transmission; and panel (D) shows the network of droplet and aerosol transmission combined. The edges for droplet transmission are always bidirectional, hence no arrows are shown. The edges for aerosol transmission may be unidirectional due to the temporal delay of virus shedding and virus uptake, hence arrows are shown.

**Figure 2:**
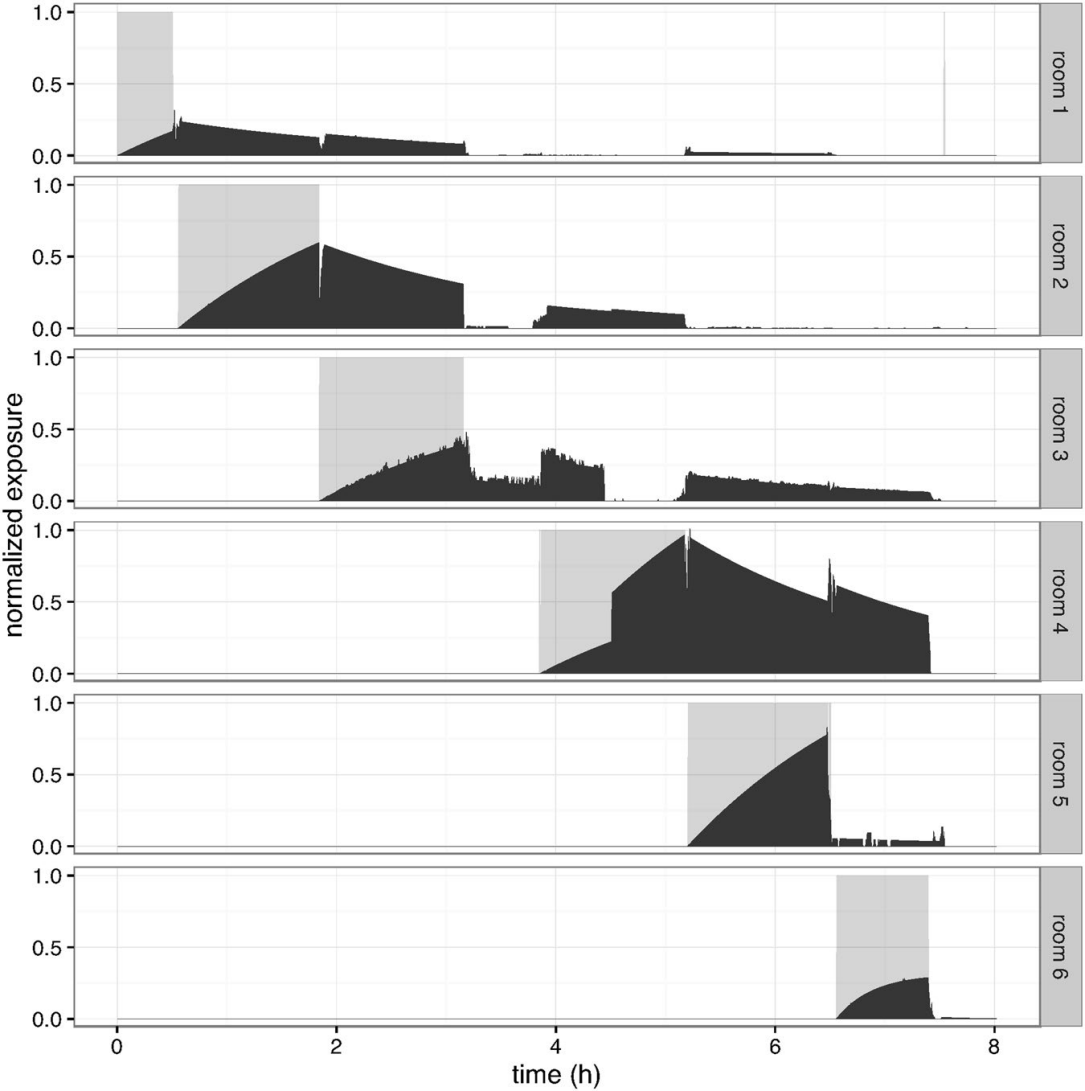
Illustration of relationship between presence of infected individual and exposure to others in the aerosol model. Presence of one (selected) infected individual in different rooms (gray area) and other people’s exposure to infectious material shed by the individual in the respective rooms (black bars) across 8 hours; the scale of the x-axis is hours, the scale of the y-axis is exposure in [0–0.02] quanta. Exposure levels depend on the number of exposed individuals in the room following deposition of infectious material, as well as air change rates (here 0.5).

We first investigate the dynamics of influenza spread in three different transmission models, namely a droplet-based, an aerosol-based, and a combined droplet-aerosol-based model (for a schematic explanation of these transmission models, please see Fig. 1). These three models were chosen to compare the two extreme situations (droplet-only, and aerosol-only) as well as an intermediate situation where the two transmission modes are equally relevant^13,14^. Simulations of influenza outbreaks were based on an SEIR model run on a high-resolution contact network collected at a US high school^17^ using wireless sensor network technology. In addition, we used the location information of each individual obtained using the same technology. In order to simulate partial or full aerosol transmission, we combined this data with building data from the school in order to compute the relevant infection probabilities due to aerosol transmission as per eq. 3. The entire model, and the data sources, are described in full detail in the Methods. Figure 2 shows an example of quanta concentrations in multiple classrooms during a day.

**Figure 3:**
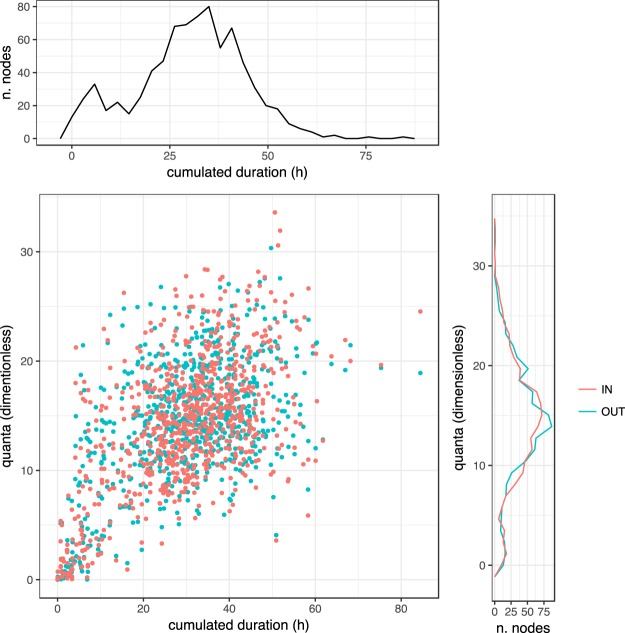
Weighted network representations of droplet transmission (cumulated contact duration) and aerosol transmission (cumulated quanta exposure). Quanta exposure caused in others represents outdegree, quanta exposure to the individual of interest represents indegree. Contact duration in droplet transmission is symmetric, therefore there is no difference between in- and outdegrees. Top figure and right figure illustrate weighted degree distributions; the scatter plot relates network metrics for droplet and aerosol transmission to each other.

As illustrated in Fig. 1, both droplet and aerosol transmission can be represented as weighted networks. It has been shown previously that strength (i.e., the weighted degree) is a key network metric for understanding disease spread in networks: hosts with a high strength generate on average more secondary cases, hosts with a low strength less^18–20^. Figure 3 shows distribution of and correlation between measures of strength for both modes of transmission, aerosol (quanta) and droplet (contact duration). As can be seen, distributions are similar and both strength measures are correlated across modes of transmission.

For all three transmission models, we measure three epidemiological quantities: the final size of an outbreak (number of recovered *r*(*t*) individuals after a simulation run), the total duration of an outbreak (time until there are no more exposed *e*(*t*) or infected *i*(*t*) individuals, respectively), and the time to reach the peak of the outbreak, i.e. to reach the maximal prevalence. In addition, in order to be able to put these quantities in context, we also measure *R*_0_ for the three transmission models. In an individual-based model like the one used here, measuring *R*_0_ is straightforward using the droplet-based transmission model, because one can directly track which individual infected which. However, in the case of partial or full aerosol-mediated transmission where infection is mediated by the air in a room, this tracking is harder because one would need to computationally keep track of the individual sources of infectious aerosol particles. We thus measure an alternative but similar quantity, namely the number of all cases infected during the initial time period until the index case recovers. We call this quantity *R*′_0_. Theoretically, there is a chance that this overestimates the true *R*_0_ by including third generation cases that were not infected by the index case, but given the values for the incubation period and the recovery rate, this is rather rare.

**Figure 4:**
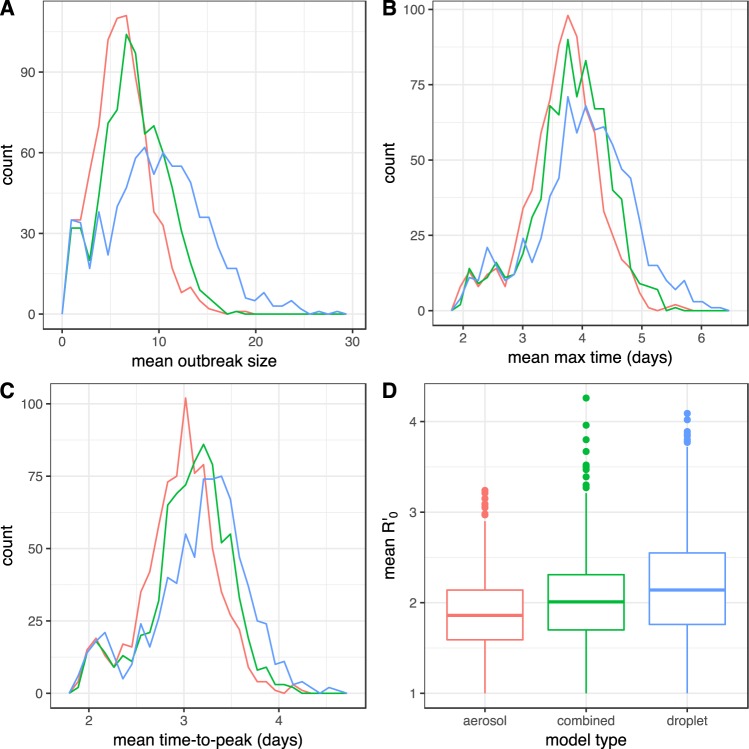
Frequency distributions of mean final size (**A**), mean duration of outbreak (**B**) mean time to reach the maximum prevalence (**C**), and mean *R*′_0_ (**D**), for the three different transmission models. Color-codes in panels A, B and C follow the labels on the x-axis in panel D (red for aerosol-based, green for combined, and blue for droplet-based transmission). For each transmission model, the values are based on 78,900 simulations where each individual served as index case in 100 independent simulation runs.

In Fig. 4, we can observe that increasing the relative importance of aerosol-based transmission (i.e., shifting from pure close-contact transmission via combined to pure aerosol-based transmission) has no major effect overall on disease dynamics. We note that outbreak sizes in the pure droplet model are slightly increased, and the time to outbreak peak is slightly increased. However, the small increase in outbreak size is also reflected in a small increase of *R*′_0_ (see Fig. 4D). In summary, our results indicate that one should not necessarily expect influenza disease dynamics to be very different when taking aerosol-based transmission into account.

**Figure 5:**
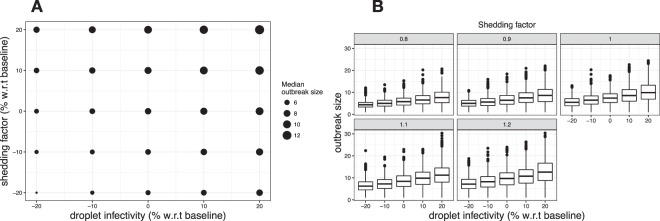
Sensitivity plots showing the effect of changes in relevant transmission parameters on outbreak size. In particular, both shedding rate and droplet infectivity were shifted from −20% up to +20% with respect to baseline values, as reported in the literature. (**A**) Scatter plot summarizing the effect of both parameters changes on median outbreak size (proportional to circles size, as shown in legend). (**B**) Boxplots plot presenting the details on the outbreak size distributions, depending on changes in droplet infectivity (x axis) and shedding rate (horizontal facets).

For a better understanding of the behavior of the transmission models, we performed a sensitivity analysis on the core infectivity parameters (Fig. 5). In particular, we altered the droplet infectivity in the close-contact component (baseline set to 0.003 – see eq. 1) and the shedding rate (*q* – see eq. 2) in the aerosol component within the model that captures both modes of transmission at 50%. We changed both parameters, relative to the baselines, by −20%, −10%, 0%, 10% and 20%. As can be seen in Fig. 5, changes on median outbreak size appear to be well-behaved and proportionate, regardless whether parameters for close-contact or aerosol transmission are changed.

**Figure 6:**
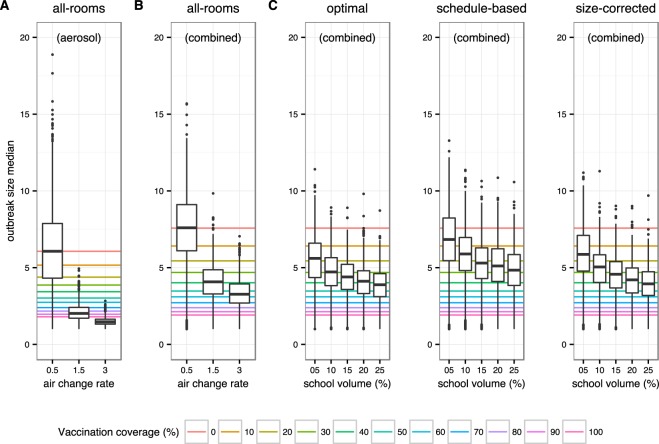
Comparison of the effect of ventilation (boxplots) and vaccination coverage (horizontal lines) on outbreak size. Colors refer to different vaccination coverages. Results are reported for aerosol-based (**A**) and combined transmission models (**B**,**C**). For (**A**,**B**), air change rate varies from 0.5 to 3.0 changes/h, while (**C**) assumes an air change rate of 0.5 changes/h. (**C**) Comparison of partially improved ventilation strategies (boxplots) in the school. Classrooms for improved ventilation were selected by ranking them according to the amount of inhaled infectious particles (optimal), their occupancy according to the school roster (schedule-based), or occupancy corrected by room size (size-corrected). Effect of vaccination coverage is also reported (median outbreak sizes under corresponding vaccination coverages, reported as horizontal lines).

In pure droplet-based transmission models, vaccination is a powerful strategy to mitigate the spread of an infectious disease. When transmission can also be aerosol-based, increasing ventilation is an additional way to curb the spread of disease. We therefore compared the effect of ventilation to traditional vaccination strategies. According to the American Society of Heating, Refrigerating and Air Conditioning Engineers (ASHRAE)^21^, a good ventilation in classrooms corresponds to 3 air changes per hour. Most classrooms, however, have poor ventilation at rates around 0.5 air changes per hour (see Methods for more details). In the pure aerosol-based model, bringing all rooms to the recommended ventilation rate would almost completely eliminate the chance of an outbreak. This corresponds to the same effect of complete vaccination coverage in the case of poor ventilation rates, as shown in Fig. 6B. In the combined droplet-aerosol scenario, improvements of ventilation still results in a significant decrease of outbreak sizes. In particular, Fig. 6A shows that in the combined droplet-aerosol model, a good ventilation would have a similar effect to a 50–60% vaccination coverage in the poor ventilation scenario. This finding proved to be robust to changes in transmission parameters in the sensitivity analysis (data not shown).

In practice, upgrading the ventilation system of an entire school campus to the rates proposed by ASHRAE will often be challenging due to limited resources. We therefore asked how strong the mitigating effect would be of upgrading the ventilation of only a fraction of all rooms. We also asked how one would identify the optimal set of rooms for mitigation purposes. The room selection strategies we explore are *optimal*, *schedule-based*, and *size-corrected*, which are all described in the methods. As expected, applying good ventilation to less rooms instead of the entire school leads to less pronounced improvements. However, Fig. 6C shows that selecting only a fraction of rooms for improved ventilation, according to the criteria explained above, still results in median outbreak sizes comparable with those obtained in a setting with 30–40% vaccination coverage. In particular, the *size-corrected* strategy, which requires only information readily available to each school (i.e. school rosters and room size) can result in median outbreak sizes that are comparable to those obtained with vaccination rates above 40%, even when only applied to 25% of all rooms.

Comparisons between the effects of improved ventilation and vaccination also depend on vaccine efficacy. The baseline efficacy was assumed to be 60%. If the ASHRAE recommended air change rate were implemented school-wide, this would result in a protective effect in the combined model that would correspond to a vaccination coverage of 60–70% for an efficacy of 40%, 50–60% for 60% efficacy, and 40–50% for 80% efficacy. In the aerosol model, consistently improved ventilation beats vaccination even with full coverage if efficacies are low.

## Discussion

There is mounting evidence that aerosol-transmission is an important factor in the spread of influenza^8–14^. Despite this, virtually all infectious disease dynamics models on influenza have thus far ignored aerosol-transmission. Here, we parameterized a model with empirically obtained values to investigate the dynamics and control of influenza under the assumptions of no, partial, or full aerosol transmission. In order to create a realistic model, we used contact network and location data that was previously obtained at a US high school using wireless sensor network technology^17^. This dataset is well-suited for the objective of the study: modeling droplet-based transmission requires data on close proximity contacts, and modeling aerosol-transmission requires data on location in rooms and information about the rooms, such as the room size. The dataset used here, even though limited in scope, and particularly also in duration (data were of only one day and had to be used repeatedly, thus exaggerating correlation between school days), contains all of this information.

Using empirical estimates of various influenza-related parameters, we found that the overall disease dynamics does not differ substantially between the models using no, partial, or full aerosol transmission. This isn’t entirely surprising, given the fact that aerosol transmission parameters were estimated from the same influenza outbreak that was used to parameterize previous (pure droplet-based) influenza models. However, aerosol transmission does change the underlying transmission network (see Fig. 1), which in turn could nevertheless have a substantial impact on disease dynamics, depending on the specific co-location patterns of individuals. Our finding that the dynamics do not change substantially in a model parameterized by empirical co-location data may simply be a reflection of the fact that schools are high density environments with comparatively limited movement, and - in line with this - we found that the underlying transmission-dependent network structures to be very similar with respect to the key network property strength. It should also be noted that we did not assume any virus inactivation in our model, largely because it is generally known to be slow for low relative humidity values (typical for indoor air during influenza season)^6,7,22^. Nevertheless, this assumption may overestimate the relative effectiveness of ventilation somewhat.

While vaccination is at the heart of influenza prevention efforts, aerosol-based transmission of influenza opens up additional possibilities to control the spread of the disease. In particular, when infectious agents can remain airborne, air ventilation is a well known method to mitigate disease spread^23^. In this study, we assessed potential effects of bringing the air change rates up to the recommended levels by the American Society of Heating, Refrigerating and Air Conditioning Engineers (ASHRAE)^21^, which defines an acceptable ventilation in classrooms to be 3 air changes per hour. We found that by doing so, we were able to generate reductions in expected outbreak sizes that would normally only be possible with a substantial vaccination coverage of 50–60%, which is within the range of observed vaccination rates in school settings^24^. Moreover, even when bringing only a quarter of the rooms to the recommended air change rates, using easy-to-obtain data in order to select the best rooms, we were still able to obtain outbreak size reductions that would require 30–40% vaccination coverage when air change rates are at levels commonly reported at US schools. The concrete percentages, obviously, depend on modeling assumptions as well as transmission parameters.

Our results suggest that improvements of ventilation in high density public spaces could be an important and relatively easy-to-implement strategy supplementing vaccination efforts for effective control of influenza spread. This observation rests on the assumption that at a substantial part of influenza spread is due to aerosol-based transmission, for which there is mounting evidence. Given that increased air ventilation rates are not known to have any negative side effects, and that there are numerous infectious diseases that are entirely or partially transmitted via aerosol (e.g. tuberculosis), the findings here thus provide an additional argument corroborating the public health recommendations for good air ventilation. It should be noted that influenza vaccine effectiveness is often less than the here assumed 60%^25,26^, whereas good ventilation would provide increased protection, further underlining its importance.

## Methods

### Data

The data used in this paper were collected at a US high school during one school day using wireless sensor technology. In total, 789 individuals (94% of the school population) participated. They wore small sensors that detect and record radio signals broadcast by other nearby sensors. Further, stationary devices broadcasting signals were attached to fixed locations (at least one per room) throughout the school campus to keep track of the participants’ locations.

Consequently, the data include two types of records. Close proximity interactions (CPIs) are records that indicate two participating individuals standing face-to-face with a distance of less than three meters at a certain point in time. Location records are records that indicate the presence of an individual nearby a stationary device (location information is at the level of rooms).

A detailed description on how information and noise were separated in the data is provided elsewhere^18^. Data were collected at time intervals of 20 seconds.

### Model of influenza spread

We used an individual-based model with a susceptible, exposed, infectious, recovered (SEIR)-type structure. We assumed that influenza is introduced into the school population by one index case at the beginning of a simulation run and that no further introductions from outside occur. The duration of a simulation time step was half a day (i.e., contact information was aggregated at this level, which was shown to be a reasonable approximation for full-resolution networks^27^; also note that the temporal resolution of the model of virus particle air concentrations was kept at 20 seconds and only exposure levels were aggregated at the this level, see also below). Infection transmission could only occur during the half-day including school, not during the half-day including the night. Individual *j*’s probability *P*_*j*_ to switch from the susceptible to the exposed state depends on the mode of transmission. We defined one function *P*_*a*,*j*_ for aerosol transmission and one function *P*_*cc*,*j*_ for close-contact transmission, as laid out below. We ran simulations for an all-aerosol scenario, for an all-close-contact scenario, and one for a scenario where both aerosol and close-contact transmission occur as 0.5*P*_*a*,*j*_ + 0.5*P*_*cc*,*j*_. The duration of the exposed state follows a Weibull distribution with an offset of half a day; the power parameter is 2.21, the scale parameter is 1.10^17,28^). After that period in the exposed state, every individual will be in the infectious state for exactly one time step before turning into home confinement and, finally, recovering. To allow for the fact that the onset of influenza symptoms is typically sudden and that affected individuals will be dismissed quickly, we reduced *P*_*j*_ by 75%, as described in Salathé *et al*.^17^, which also contains more details on all parameter choices other than those specific to the aerosol transmission probability.

#### Close-contact transmission probability

Assumptions, parameters, and structure of the close-contact transmission model are described in detail elsewhere^17^, and therefore only described briefly here.

Close-contact transmission requires social interaction between an infectious and a susceptible individual, and it includes transmission via large droplets that do not travel far and do not stay suspended in the indoor air as well as transmission via direct, physical contact^29^. Risk of transmission is usually operationalized as a function of contact duration^30^. Based on data from an outbreak on a commercial airliner^31^, the probability of transmission was estimated as

1 $${P}_{cc,j}=1-{(1-0.003)}^{T}$$

with *T* being the contact duration between two individuals in number of sensor recordings (every 20 s)^17^.

#### Aerosol transmission probability

In our model, we quantify amounts of aerosolized virus particles in ‘quanta’, following Wells’^32^ quantum theory of disease transmission. A quantum is defined as the amount of infectious droplet nuclei required to infect the fraction 1 − 1/*e* of a susceptible population exposed to it.

We assume that every room of the school is a well-mixed airspace that is only connected to the outside, but does not exchange air with other rooms. We further assume that removal of aerosolized virus particles by ventilation is the dominant removal process and that, e.g., neither inactivation nor settling play an important role at low levels of relative humidity typical for influenza season (which is a standard assumption in the literature, cf., e.g.^22,33^). Under these assumptions, we model the concentration of virus particles in a particular room *r* as

2 $$\frac{{\rm{\Delta }}{C}_{r,t}}{{\rm{\Delta }}t}=\frac{{\sum }_{i\in {I}_{r,t}}{q}_{i,t}}{{V}_{r}}-{C}_{r,t}\frac{{Q}_{r,t}}{{V}_{r}}$$

where *C*_*r*,*t*_ is the quanta concentration in room *r* at time *t*, *I*_*r*,*t*_ is the set of all infectious individuals that are in room *r* at time *t*, *q*_*i*,*t*_ is the quanta shedding rate of infector *i* at time *t*, *V*_*r*_ is the volume of room *r*, and *Q*_*r*,*t*_ is the fresh air supply rate of room *r* at time *t*. The quotient *Q*_*r*,*t*_/*V*_*r*_ is also known as the air change rate (ACR).

The instantaneous dose of infectious material, *D*_*j*,*t*_, inhaled by individual *j* at time step *t* is given by

$${D}_{j,t}={C}_{r,t}{p}_{j}{\rm{\Delta }}t$$

where *C*_*r*,*t*_ is the quanta concentration in room *r* - the room in which individual *j* is located at time *t* - at time *t*, *p*_*j*_ is the breathing rate of individual *j*, and Δ*t* is the duration of a simulation time step, here 20 s.

Individual *j*’s total exposure, *D*_*j*_ during an entire school day is given by

$${D}_{j}={\sum }_{t={t}_{0}}^{{t}_{x}}{D}_{j,t}.$$

Combining the total daily exposure with Wells’ definition of quanta allows to model the probability *P*_*a*,*j*_ of a fully susceptible individual to become infected during one simulation school day as

3 $${P}_{a,j}=1-\exp (\,-\,{D}_{j})$$

where the total exposure *D*_*j*_ is the only parameter required.

Shedding rate: Both bottom-up^3,34^ and top-down approaches^35^ have been used by others to estimate quanta-based shedding rates for influenza. Bottom-up studies (mechanistic approaches starting from basic measurements and processes) suffered from huge uncertainties and differed by three orders of magnitude. We used data from Rudnick and Milton’s^35^ top-down study that back-calculated quanta shedding rates from the same outbreak data^31^ that was also used to parameterize the close-contact model^17^. They estimated shedding rates of between 79 quanta/h and 128 quanta/h, depending on model assumptions. We chose a shedding rate of 100 quanta/h for our aerosol transmission model.

Ventilation rate: We assumed different scenarios for the ventilation rate. According to the ventilation recommendations for schools by the American Society of Heating, Refrigerating and Air Conditioning Engineers (ASHRAE)^21^, the ventilation rate for classrooms should be at least 8 l/s per person. Daisey *et al*.^36^ estimate that for a typical classroom situation, this corresponds to an air change rate (ACR) of 3.0 air changes per hour. This estimate served as our good-ventilation scenario. Various studies found substantially lower ACR in US schools^36–38^, and CO_2_ concentrations at the high school we collaborated with (unpublished data) indicated very poor ventilation conditions, too. In line with reported ACR in other US schools, we assumed 0.5 air changes per hour for a poor ventilation scenario. Additionally, we used 1.5 air changes per hour as a middle scenario.

Breathing rate: The breathing rate of humans depends mainly on their age, gender, and activity levels^39^. In line with Adams’^39^ measurements and in accordance with other work in the field^35^, we assumed a constant breathing rate of 8 l/min for every individual.

#### Interventions

We compared ventilation-based interventions (effect only on aerosol transmission) with vaccinating individuals (effect independent on transmission pathway).

Ventilation rate: Baseline scenario to which all intervention scenarios were compared with was the poor ventilation scenario (ACR 0.5 h^−1^). Basic interventions were improving the ventilation to ASHRAE standards (ACR 3.0 h^−1^) and to achieve an intermediate improvement (ACR 1.5 h^−1^), respectively.

We further analyzed how ventilation improvement only in some rooms would affect infection spread. We defined three different methods to identify rooms for which the ACR was increased from 0.5 h^−1^ to 3.0 h^−1^: (i) *optimal*, using all available information from simulation runs, we identified rooms with the highest cumulative exposure, i.e., where most susceptibles will be exposed or where doses are highest in a typical simulation run; (ii) *schedule-based*, identifying rooms with the highest cumulative occupancy throughout a school day according to the school’s official roster; (iii) *size-corrected*, which is similar to the schedule-based approach, but the total occupancy was divided by the volume of the room to give priority to small rooms with a high occupancy, as quanta concentration builds up faster in small rooms.

All three methods were used to identify rooms that represent 5%, 10%, 15%, 20% and 25% of the total indoor space. Methods (ii) and (iii) could realistically be applied in a school setting. Comparing interventions based on them with optimal ones allows assessing their relative performance to the theoretical optimum.

Vaccination: We assumed a vaccination effectiveness (protection against transmission) of 60%^26^ as a standard scenario (In sensitivity analyses we also assumed 40% and 80%) and a random distribution of vaccination status among the school population. We simulated the impact of vaccination for vaccination coverage values between 0% (baseline) and 100% with increments of 10%.

### Ethical approval

All measurements involving human subjects were conducted according to the relevant regulations and involved an informed consent obtained from all subjects. The whole study was previously approved by the Stanford IRB (Institutional Review Board).

### Accession codes

The data code to reproduce results and figures are available on a GitHub repository at https://github.com/salathegroup/aerosol. The input files needed to reproduce the computer simulations can be found in a dedicated folder at https://github.com/salathegroup/aerosol/tree/master/input_files.

## References

[1] Brankston G, Gitterman L, Hirji Z, Lemieux C, Gardam M (2007). Transmission of influenza a in human beings. The Lancet infectious diseases.

[2] Killingley B, Nguyen-Van-Tam J (2013). Routes of influenza transmission. Influ. other respiratory viruses.

[3] Fabian P (2008). Influenza virus in human exhaled breath: an observational study. PloS one.

[4] Gralton J, Tovey E, McLaws M-L, Rawlinson WD (2011). The role of particle size in aerosolised pathogen transmission: a review. J. Infect..

[5] Stilianakis, N. I. & Drossinos, Y. Dynamics of infectious disease transmission by inhalable respiratory droplets. *J. Royal Soc. Interface* rsif20100026 (2010). 10.1098/rsif.2010.0026 PMC2894888 20164087

[6] Weber TP, Stilianakis NI (2008). Inactivation of influenza a viruses in the environment and modes of transmission: a critical review. J. infection.

[7] Tellier R (2006). Review of aerosol transmission of influenza a virus. Emerg Infect Dis.

[8] Atkinson MP, Wein LM (2008). Quantifying the routes of transmission for pandemic influenza. Bull. mathematical biology.

[9] Tellier, R. Aerosol transmission of influenza a virus: a review of new studies. *J. Royal Soc. Interface* rsif20090302 (2009). 10.1098/rsif.2009.0302.focus PMC2843947 19773292

[10] Mubareka S (2009). Transmission of influenza virus via aerosols and fomites in the guinea pig model. J. Infect. Dis..

[11] Wong BC (2010). Possible role of aerosol transmission in a hospital outbreak of influenza. Clin. infectious diseases.

[12] Noti, J. D. *et al*. Detection of infectious influenza virus in cough aerosols generated in a simulated patient examination room. Clin. Infect. *Dis*. cis237 (2012). 10.1093/cid/cis237 PMC4680957 22460981

[13] Cowling, B. J. *et al*. Aerosol transmission is an important mode of influenza a virus spread. *Nat. communications* **4** (2013). 10.1038/ncomms2922 PMC3682679 23736803

[14] Lau MS, Cowling BJ, Cook AR, Riley S (2015). Inferring influenza dynamics and control in households. Proc. Natl. Acad. Sci..

[15] Xiao, S. *et al*. Probable transmission routes of the influenza virus in a nosocomial outbreak. *Epidemiol. & Infect*. 1–9 (2018). 10.1017/S0950268818001012 PMC9134375 29729675

[16] Lindsley, W. G. *et al*. Viable influenza a virus in airborne particles expelled during coughs versus exhalations. *Influ. other respiratory viruses* (2016). 10.1111/irv.12390 PMC4947941 26991074

[17] Salathé M (2010). A high-resolution human contact network for infectious disease transmission. Proc. Natl. Acad. Sci..

[18] Smieszek T, Salathé M (2013). A low-cost method to assess the epidemiological importance of individuals in controlling infectious disease outbreaks. BMC Medicine.

[19] Bell D, Atkinson J (1999). Centrality measures for disease transmission networks. Soc. Networks.

[20] Christley R (2005). Infection in social networks: using network analysis to identify high-risk individuals. Am. J. Epidemiol..

[21] American Society of Heating, Refrigerating and Air-Conditioning Engineers. *ANSI/ASHRAE Standard 62.1-2016: Ventilation for Acceptable Indoor Air Quality* (American Society of Heating, Refrigerating and Air-Conditioning Engineers, 2016). PMC5514843 28729740

[22] Yang W, Marr LC (2011). Dynamics of airborne influenza a viruses indoors and dependence on humidity. PLOS One.

[23] Li Y (2007). Role of ventilation in airborne transmission of infectious agents in the built environment–a multidisciplinary systematic review. Indoor air.

[24] Barclay VC (2014). Positive network assortativity of influenza vaccination at a high school: implications for outbreak risk and herd immunity. PloS one.

[25] Belongia EA (2016). Variable influenza vaccine effectiveness by subtype: a systematic review and meta-analysis of test-negative design studies. The Lancet Infect. Dis..

[26] Osterholm MT, Kelley NS, Sommer A, Belongia EA (2012). Efficacy and effectiveness of influenza vaccines: a systematic review and meta-analysis. The Lancet infectious diseases.

[27] Stehlé J (2011). Simulation of an seir infectious disease model on the dynamic contact network of conference attendees. BMC Medicine.

[28] Ferguson NM (2005). Strategies for containing an emerging influenza pandemic in southeast asia. Nat..

[29] Smieszek T (2014). How should social mixing be measured: comparing web-based survey and sensor-based methods. BMC infectious diseases.

[30] Smieszek T (2009). A mechanistic model of infection: why duration and intensity of contacts should be included in models of disease spread. Theor. Biol. & Med. Model..

[31] Moser MR (1979). An outbreak of influenza aboard a commercial airliner. Am. J. Epidemiol..

[32] Wells, W. F. *Airborne contagion and air hygiene. An ecological study of droplet infections* (Harvard University Press, Cambridge, MA, 1955).

[33] Azimi P, Stephens B (2013). Hvac filtration for controlling infectious airborne disease transmission in indoor environments: Predicting risk reductions and operational costs. Build. Environ..

[34] Chen S-C, Liao C-M (2010). Probabilistic indoor transmission modeling for influenza (sub) type viruses. J. Infect..

[35] Rudnick S, Milton D (2003). Risk of indoor airborne infection transmission estimated from carbon dioxide concentration. Indoor air.

[36] Daisey JM, Angell WJ, Apte MG (2003). Indoor air quality, ventilation and health symptoms in schools: an analysis of existing information. Indoor Air.

[37] Shendell DG (2004). Air concentrations of vocs in portable and traditional classrooms: results of a pilot study in los angeles county. J. Expo. Sci. Environ. Epidemiol..

[38] Mullen N, Bhangar S, Hering S, Kreisberg N, Nazaroff W (2011). Ultrafine particle concentrations and exposures in six elementary school classrooms in northern california. Indoor Air.

[39] Adams, W. Measurement of breathing rate and volume in routinely performed daily activities, final report. *Hum. Perform. Lab. Phys. Educ. Dep. Univ. California, Davis. Prep. for California Air Resour. Board, Contract*. (1993).

