# Author Correction: Assessing the Dynamics and Control of Droplet- and Aerosol-Transmitted Influenza Using an Indoor Positioning System

**DOI:** 10.1038/s41598-020-62682-9

**Published:** 2020-03-27

**Authors:** Timo Smieszek, Gianrocco Lazzari, Marcel Salathé

**Affiliations:** 1 Modelling and Economics Unit, National Infection Service, Public Health England, London, UK; 2 MRc Centre for Outbreak Analysis and Modelling, Department of Infectious Disease Epidemiology, Imperial College School of Public Health, London, UK; 3 Center for Infectious Disease Dynamics, The Pennsylvania State University, University Park, USA; 4 Global Health Institute, School of Life Sciences, Ecole Polytechnique Fédérale de Lausanne (EPFL), Lausanne, Switzerland

Correction to: *Scientific Reports* 10.1038/s41598-019-38825-y, published online 18 February 2019

This Article contains errors in the Results section where,

“This corresponds to the same effect of complete vaccination coverage in the case of poor ventilation rates, as shown in Fig. 6B. In the combined droplet-aerosol scenario, improvements of ventilation still results in a significant decrease of outbreak sizes. In particular, Fig. 6 A shows that in the combined droplet-aerosol model, a good ventilation would have a similar effect to a 50–60% vaccination coverage in the poor ventilation scenario.”

should read:

“This corresponds to the same effect of complete vaccination coverage in the case of poor ventilation rates, as shown in Fig. 6 A. In the combined droplet-aerosol scenario, improvements of ventilation still result in a significant decrease of outbreak sizes. In particular, Fig. 6B shows that in the combined droplet-aerosol model, a good ventilation would have a similar effect to a 50–60% vaccination coverage in the poor ventilation scenario.”

